# Supplementation with Oral Vitamin C Prior to and during Myeloablative Chemotherapy and Autologous Haematopoietic Stem Cell Transplantation: A Pilot Study

**DOI:** 10.3390/antiox11101949

**Published:** 2022-09-29

**Authors:** Anitra C. Carr, Emma Vlasiuk, Masuma Zawari, Natalie Meijer, Carolyn Lauren, Sean MacPherson, Jonathan Williman, Stephen T. Chambers

**Affiliations:** 1 Nutrition in Medicine Research Group, Department of Pathology and Biomedical Science, University of Otago, Christchurch 8011, New Zealand; 2 Department of Haematology, Christchurch Hospital, Christchurch 8011, New Zealand; 3 Department of Population Health, University of Otago, Christchurch 8011, New Zealand; 4 The Infection Group, Department of Pathology and Biomedical Science, University of Otago, Christchurch 8011, New Zealand

**Keywords:** vitamin C, ascorbic acid, chemotherapy, haematopoietic stem cell transplantation, HSCT, quality of life, mucositis, EORTC QLQ

## Abstract

Chemotherapy-related side effects are common in patients undergoing myeloablative chemotherapy and haematopoietic stem cell transplantation. Some, such as oral mucositis, are believed to be due to enhanced oxidative stress and inflammation. Vitamin C, a potent antioxidant with anti-inflammatory properties, becomes severely depleted following myeloablative chemotherapy. The aim of our study was to assess the feasibility and efficacy of oral vitamin C supplementation to restore and maintain adequate vitamin C concentrations in patients undergoing myeloablative chemotherapy and stem cell transplantation. We carried out a pilot randomized controlled trial in 20 patients with myeloma and lymphoma. Placebo or vitamin C tablets (1 g twice daily) were initiated one week prior to transplantation and continued for 4 weeks post-transplantation. Blood samples were collected weekly for analysis of plasma vitamin C concentrations using high-performance liquid chromatography. The patients’ symptoms and quality of life parameters were monitored using the World Health Organization oral toxicity scale and the European Organization for the Research and Treatment of Cancer Quality of Life Questionnaire (EORTC QLQ). Pre-supplementation with oral vitamin C doubled vitamin C concentrations relative to placebo by day 0 (median 61 vs. 31 µmol/L), with 60% of those in the vitamin C group achieving concentrations ≥ 50 µmol/L, compared with only 10% in the placebo group. Following chemotherapy and transplantation, significance between the vitamin C and placebo groups was lost by day 7, with only 30% of the patients in the vitamin C group having plasma concentrations ≥ 50 µmol/L. This was partly due to intolerance of the oral intervention due to nausea/vomiting and diarrhoea (40% of the participants in each group). Oral mucositis was also observed in 40% of the participants at day 7 or 14. Overall, our study showed that whilst short-term oral vitamin C pre-supplementation was able to restore adequate vitamin C status by day 0, ongoing supplementation could not maintain adequate vitamin C concentrations following chemotherapy and transplantation. Thus, intravenous vitamin C should be trialled as this bypasses the gastrointestinal system, negating intolerance issues and improving bioavailability of the vitamin.

## 1. Introduction

Patients with refractory haematopoietic malignancies, such as myeloma and lymphoma, typically undergo autologous haematopoietic stem cell transplantation. This process involves harvest and storage of haematopoietic stem cells before the patient receives high-dose myeloablative chemotherapy, +/− radiotherapy, to destroy their malignant cell population [1]. The patient’s stem cells are then reinfused to rescue the ablated marrow and restore normal haematopoiesis within a few weeks. Haematopoietic stem cell transplantation has major complications including increased risk of sepsis and septic shock due to insufficient numbers of leukocytes to fight infection. Oral mucositis is another common side effect of chemotherapy in which painful inflammation and ulceration of the mucous membranes can compromise oral intake [2]. Oral mucositis is thought to be initiated by generation of reactive oxygen species and oxidative stress by chemotherapeutic agents or radiation which subsequently activate the inflammatory response [3]. Meta-analysis has indicated that vitamins with antioxidant properties, such as vitamin E, may help to attenuate treatment-induced oral mucositis, however, few studies have assessed the role of vitamin C [4].

Vitamin C is an essential nutrient that has pleiotropic immune supportive roles in the body [5]. Vitamin C acts as a potent antioxidant, able to scavenge a wide range of reactive oxygen species, thus protecting cells and tissues from oxidative damage and dysfunction [6], and also has anti-inflammatory properties [7]. Thus, vitamin C’s antioxidant and anti-inflammatory functions could conceivably help attenuate the incidence and severity of chemotherapy-related side effects. In support of this notion, administration of high-dose intravenous vitamin C to oncology patients undergoing chemotherapy has been shown to decrease off-target organ toxicity [8], and decrease common cancer and chemotherapy-related symptoms such as nausea/vomiting, fatigue and pain, thereby improving patient quality of life [9]. Low-dose intravenous vitamin C has also been administered with arsenic trioxide monotherapy or combination therapy to reduce toxicity and improve tolerability of the chemotherapy for the treatment of refractory myeloma [10].

Chemotherapy can significantly deplete vitamin C concentrations in oncology patients [11], including patients undergoing haematopoietic stem cell transplantation [12,13]. We, and others, have shown that vitamin C depletion was associated with increased markers of inflammation and oxidative stress in these patients [13,14]. Patients with haematopoietic malignancies, such as myeloma, have been shown to have elevated concentrations of lipid oxidation products relative to control participants [15,16]. Supplementation with parenteral vitamin C was shown to significantly increase the vitamin C status of bone marrow transplantation recipients and also decrease the elevated markers of lipid oxidation [17]. Of note, preliminary data suggests that patients with more severe mucositis tend to have lower vitamin C status [18]. Furthermore, a small study in six patients following allogeneic stem cell transplantation showed improved vitamin C status with oral vitamin C supplementation (2 g/day), as well as marked improvement in mucous membranes after eight weeks of therapy, resulting in the patients being able to eat without pain or restriction [19].

In the current study, we aimed to determine the feasibility and efficacy of oral vitamin C supplementation at restoring and maintaining adequate vitamin C concentrations in patients undergoing myeloablative chemotherapy and stem cell transplantation. We carried out a pilot randomised controlled trial in 20 patients scheduled for autologous haematopoietic stem cell transplantation for myeloma and lymphoma. A vitamin C dose of 2 g/day was chosen as this is the upper recommended oral intake in many countries [20], and the target plasma vitamin C concentration was ≥50 µmol/L as this is considered ‘adequate’ [21]. We have previously shown that plasma vitamin C values correlate closely with tissue concentrations and hence are an indicator of body status [22]. Although not powered to detect clinically meaningful differences in clinical outcomes, we also measured chemotherapy-related side effects and quality of life impacts to generate pilot data for future trials.

## 2. Materials and Methods

### 2.1. Study Design and Participants

This was a double-blind randomized, placebo-controlled feasibility and pilot study carried out at Christchurch Hospital, New Zealand (August 2019—September 2021). The study received ethical approval from the New Zealand Central Human and Disability Ethics Committee (approval # 19CEN67) and was registered with the Australian and New Zealand Clinical Trial Registry (ACTRN12619000736145). The participants were recruited from adult patients scheduled for myeloablative chemotherapy and autologous haematopoietic stem cell transplantation for myeloma and lymphoma. Exclusion criteria included: not able to provide written consent, poor renal function (creatinine clearance < 10 mls/sec or glomerular filtration rate < 15) and haemochromatosis. None of the participants were taking vitamin C supplements. Participant demographics, diagnosis and chemotherapy regimen were recorded.

**Figure 1:**
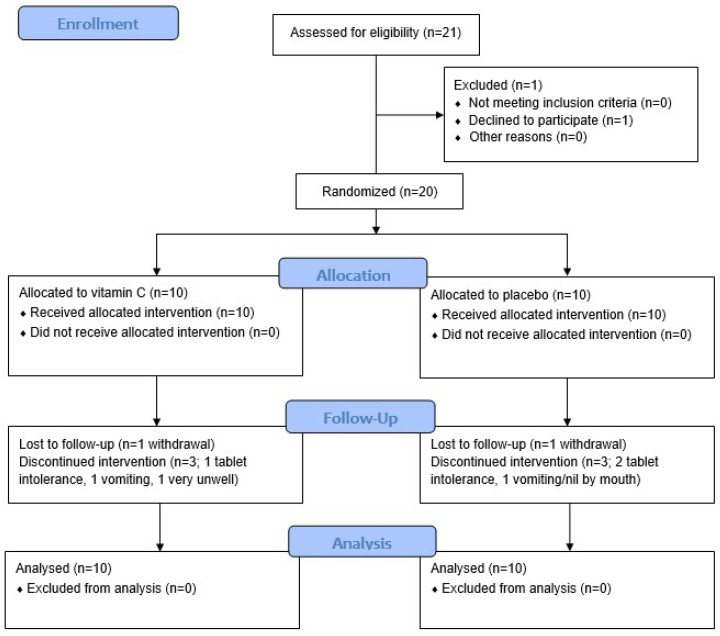
CONSORT diagram.

The primary objective of the study was to determine the proportion of participants who achieved and maintained adequate concentrations of vitamin C, defined as a minimum concentration of 50 µmol/L plasma vitamin C [21]. A sample size of 34 (17 per group) would provide 80% power at a two-sided alpha of 0.05 to detect an absolute difference of 45% in the proportion of patients maintaining optimal levels of vitamin C (from 5% in the control group, to 50% or more in the intervention group) based on data obtained from our earlier observational study [13]. The study was terminated at 20 participants because recruitment of the immune-compromised cohort was limited during the novel coronavirus pandemic and resultant nationwide lockdowns (see CONSORT diagram; Figure 1).

### 2.2. Intervention

The participants were randomised (1:1, random size blocks) to receive placebo control or 2 g/day oral vitamin C (taken as 1 g twice daily to optimise plasma concentrations [23]). Allocation was concealed using sequentially numbered opaque sealed envelopes and both clinical staff and patients were blinded as to which arm the participants were allocated. The intervention was begun 7 days prior to transplantation (day-7) and was continued for four weeks post-transplantation (if tolerated). The intervention was initially chewable tablets supplied by Tishcon Corp, Westbury, NY, USA. However, the first three participants to receive the chewable tablets could not tolerate the flavour and/or texture due to nausea/vomiting following transplantation and ceased taking the tablets from days 3–6. Therefore, capsules containing placebo powder or ascorbic acid powder (VitaFit, vitamins.co.nz, Waitakere, New Zealand) were used from the fourth participant onwards. These were well tolerated, unless the participant had ongoing vomiting following transplantation.

### 2.3. Collection and Analysis of Blood Samples

Blood samples were collected at time of stem cell harvest (baseline), day 0 (day of transplantation), and days 7, 14, 21, and 28 (post-transplantation). Samples were immediately sent to Canterbury Health Laboratories, an International Accreditation New Zealand (IANZ) laboratory, for routine haematological, biochemical and liver function tests. Vitamin C analyses were carried out by high-performance liquid chromatography [24].

### 2.4. Assessment of Side Effects and Quality of Life

Patient vital signs were recorded, and study-related questionnaires were administered at the same time points as for blood collection (baseline, days 0, 7, 14, 21, 28). The incidence and severity of oral mucositis was monitored using the World Health Organization oral toxicity scale: Grade 0, no change; Grade 1, soreness/erythema; Grade 2, erythema, ulcers, can eat solid food; Grade 3, ulcers, requires liquid diet only; Grade 4, alimentation not possible [25]. Patient symptoms and functioning were monitored using the European Organization for the Research and Treatment of Cancer Quality of Life Questionnaire (EORTC QLQ-C30) [26]. The EORTC QLQ-C30 comprises five multi-item functional scales (physical, role, cognitive, emotional, and social), three multi-item symptom scales (fatigue, pain, and nausea/vomiting), and a global health and quality of life scale. Additional single item scales include symptoms commonly reported by oncology patients (e.g., dyspnoea, appetite loss, sleep disturbance, constipation, and diarrhoea). These are scored on a 4-point Likert scale: 1, not at all; 2, a little; 3, quite a bit; 4, very much. The transformed measures range in score from 0 to 100, with a difference of 4–10 points representing a small change in quality of life, and a difference of 10–20 points representing a medium change [27]. Specific gastrointestinal and infection-related adverse events were also monitored using the Common Terminology Criteria for Adverse Events (CTCAE) version 4.02 [28].

### 2.5. Statistical Analyses

Data are presented as counts and percentages for categorical variables, and median and interquartile range (Q1, Q3) or mean and standard error of the mean (SEM) for continuous variables. Mixed-effects analyses were used to determine differences between the groups, with *p* < 0.05 signifying significance, using GraphPad Prism 9 (GraphPad, San Diego, CA, USA). Repeated measures correlation was undertaken using the R package *rmcorr* (4.1.1, R Core Team 2021, Vienna, Austria) to account for the non-independence of multiple observations within each subject [29,30].

## 3. Results

### 3.1. Participant Characteristics

The cohort comprised 20 participants (60% male, median age 55) who were undergoing myeloablative chemotherapy and haematopoietic stem cell transplantation for myeloma (70%) or lymphoma (30%). The myeloablative regimens are shown in Table 1. The participants were randomised to receive either placebo tablets or vitamin C tablets at a dose of 1 g twice daily; the characteristics of these groups are shown in Table 1. Two participants (10%) withdrew from the study: one in the placebo group due to nil by mouth by day 7, and one in the intervention group due to being very unwell from day 7.

### 3.2. Effect of Supplementation on Plasma Vitamin C Concentrations

**Figure 2:**
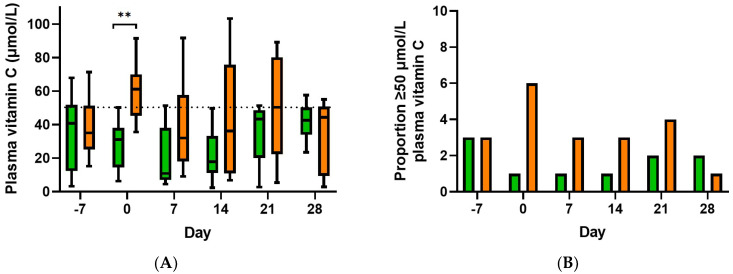
Effect of oral vitamin C supplementation on plasma vitamin C status of participants (**A**) and proportion with plasma vitamin C concentrations ≥ 50 µmol/L (**B**). Placebo group, green bars; vitamin C group, orange bars. Box plots represent median and 25th and 75th percentiles with minimum and maximum whiskers. Dashed line indicates 50 µmol/L vitamin C. Mixed-effects analysis indicated a significant difference between the two groups; ** *p* = 0.002 by post hoc analysis.

The median plasma vitamin C concentration of the participants at baseline (day of stem cell harvest) was 36 (20, 51) µmol/L. Of these, only six (30%) had concentrations ≥ 50 µmol/L, while 6 (30%) had hypovitaminosis C (concentrations ≤ 23 µmol/L) and two (10%) had outright deficiency (concentrations ≤ 11 µmol/L). Supplementation with 2 g/day vitamin C for 7 days resulted in a significant increase in plasma vitamin C concentrations relative to placebo by day 0 (61 [45, 70] µmol/L vs. 31 [15, 38] µmol/L, *p* = 0.002; Figure 2A). Of those in the vitamin C group, six (60%) had plasma concentrations ≥ 50 µmol/L, compared with only one (10%) in the placebo group (Figure 2B). However, following myeloablative chemotherapy and stem cell transplantation, significance between the vitamin C and placebo groups was lost by day 7 (32 [18, 58] µmol/L vs. 11 [7, 38], *p* = 0.099; Figure 2A). Only three (30%) of the patients in the vitamin C group had plasma vitamin C concentrations ≥ 50 µmol/L at day 7 (Figure 2B). This was partly due to intolerance of, and non-compliance with, the intervention due to nausea/vomiting and diarrhoea (four patients in each group). Supplemental Figure S1 shows changes over time as mean and SEM.

### 3.3. Infection Parameters

**Figure 3:**
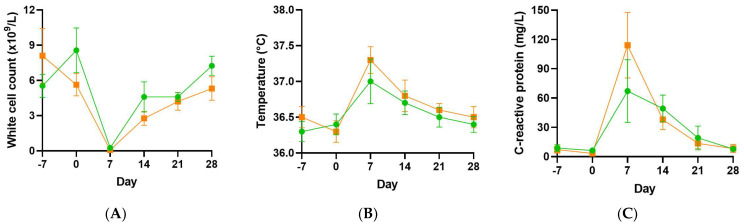
Infection parameters in the vitamin C and placebo groups. White cell counts (**A**), temperature (**B**) and C-reactive protein concentrations (**C**). Placebo group, green circles; vitamin C group, orange squares.

Following myeloablative chemotherapy and stem cell transplantation, the participants experienced febrile neutropenia as demonstrated by depleted white cell counts and elevated temperature and C-reactive protein concentrations, peaking at day 7 (Figure 3). Although the vitamin C group is shown in the figures, this pilot study was not powered to detect differences in infection parameters between the two groups.

### 3.4. Gastrointestinal Symptoms

**Figure 4:**
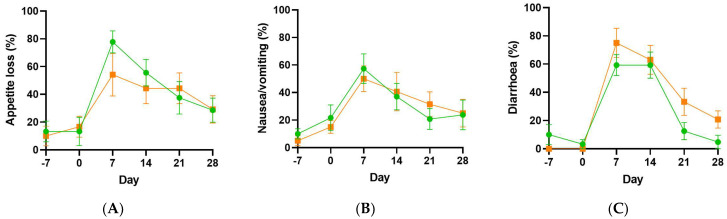
Gastrointestinal symptoms in the vitamin C and placebo groups. Appetite loss (**A**), nausea/vomiting (**B**) and diarrhoea (**C**), as determined by the EORTC QLQ. Placebo group, green circles; vitamin C group, orange squares.

By day 7, the participants were experiencing gastrointestinal disturbances, including appetite loss, nausea/vomiting and diarrhoea, as determined by the EORTC QLQ (and confirmed by CTCAE adverse event monitoring; Figure 4). Eight participants (40%) experienced oral mucositis at days 7 or 14, four in the placebo group and four in the vitamin C group. All four participants in the vitamin C group had grade 1 oral mucositis, while two of the four participants in the placebo group had grade 1 oral mucositis and the other two had grade 2 oral mucositis.

### 3.5. Health-Related Quality of Life

**Figure 5:**
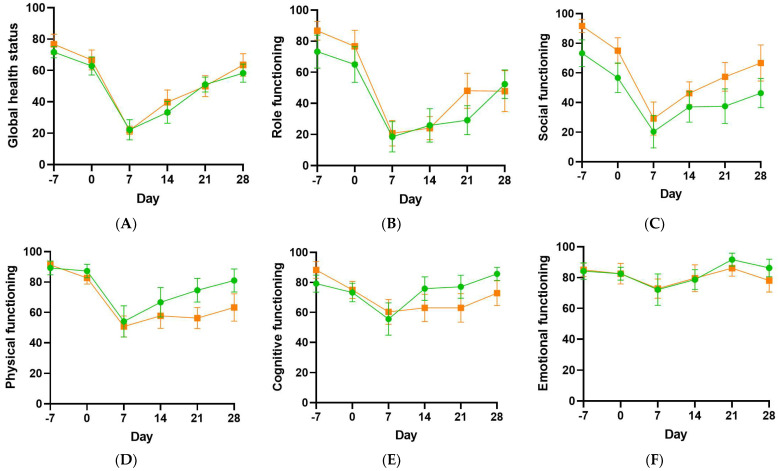

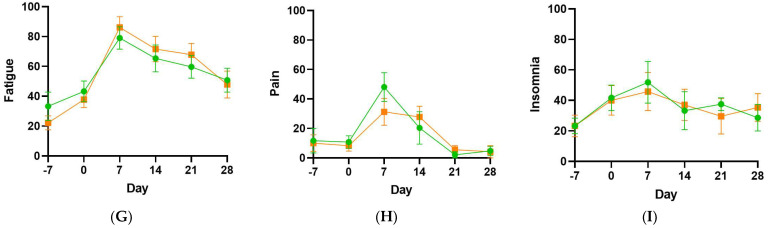
Functioning and selected symptoms in the vitamin C and placebo groups. Global health status (**A**), role functioning (**B**), social functioning (**C**), physical functioning (**D**), cognitive functioning (**E**), emotional functioning (**F**), fatigue (**G**), pain (**H**), insomnia (**I**), as determined by the EORTC QLQ. Placebo group, green circles; vitamin C group, orange squares.

Other symptoms, such as fatigue, pain and insomnia, and functioning (role, social, physical, cognitive and emotional) were assessed using the EORTC QLQ. There were dramatic drops in global health status and role and social functioning, as well as smaller decreases in physical and cognitive functioning by day 7 (Figure 5). Concurrently, there were increases in the symptoms of fatigue and pain by day 7 (Figure 5). Patient functioning and symptoms had returned to baseline values by day 28 in most categories. The study was not powered to detect differences in quality of life outcomes between the placebo and vitamin C groups.

### 3.6. Association of Plasma Vitamin C Concentrations with Post-Transplant Symptoms

Since oral vitamin C supplementation was unable to provide a statistically significant increase in vitamin C status post-transplantation relative to the placebo group, we combined the data from the two groups and investigated associations between the vitamin C status of the patients and post-transplantation symptoms and functioning. We were particularly interested in samples that achieved ≥ 50 µmol/L vitamin C (considered ‘adequate’) relative to those containing < 23 µmol/L vitamin C (hypovitaminosis C). Of the infection parameters, C-reactive protein was inversely correlated with vitamin C status in the post-transplantation samples (r = −0.53, *p* < 0.001, *n* = 60), with median C-reactive protein values of 10 (4, 19) mg/L in the samples containing ≥ 50 µmol/L vitamin C and 47 (14, 123) mg/L in the samples containing < 23 µmol/L vitamin C (Table 2).

Of the gastrointestinal symptoms, two were inversely associated with vitamin C status in the post-transplantation samples: nausea/vomiting (r = −0.52, *p* < 0.001), and diarrhoea (r = −0.53, *p* < 0.001). Of these symptoms, the ≥50 µmol/L vitamin C subgroups had significantly lower scores than the <23 µmol/L vitamin C subgroups. Other symptoms, such as fatigue and pain also showed inverse correlations with vitamin C status (Table 2). Several functional scores showed positive correlations with plasma vitamin C status; these included global health status and role and physical functioning, with social functioning showing a trend towards significance (Table 2). Of these, the ≥50 µmol/L vitamin C subgroups had significantly higher scores than the <23 µmol/L vitamin C subgroups.

## 4. Discussion

In our study of oral vitamin C supplementation of patients undergoing myeloablative chemotherapy and haematopoietic stem cell transplantation, we found that one week of supplementation (at 1 g twice daily) prior to chemotherapy and transplantation resulted in a doubling of plasma vitamin C concentrations relative to the placebo group (median 61 vs. 31 µmol/L), with 60% of those in the vitamin C group achieving plasma concentrations ≥ 50 µmol/L, compared with only 10% in the placebo group. However, following myeloablative chemotherapy and stem cell transplantation, significance between the vitamin C and placebo groups was lost by day 7, with only 30% of the patients in the vitamin C group having plasma vitamin C concentrations ≥ 50 µmol/L. This was partly due to intolerance of the oral intervention due to nausea/vomiting and diarrhoea (40% of the participants in each group). We found that the first few participants were not able to tolerate the flavoured chewable vitamin C tablets, which were subsequently changed to capsules for the remainder of the participants. Although tolerated better, inability to consume/retain the supplement due to severe or ongoing vomiting, oral mucositis or diarrhoea is a feasibility issue for oral supplementation and indicates intravenous administration of vitamin C may be required for this patient group.

Another rationale for the loss of difference in plasma vitamin C concentrations between the vitamin C and placebo groups could be the elevated inflammation and oxidative stress observed following myeloablative chemotherapy [13,14,15,16], supported by the significant inverse association between plasma vitamin C and C-reactive protein in the current study. Although administration of intravenous vitamin C has previously been shown to significantly restore the vitamin C status of bone marrow transplantation recipients, as well as decrease markers of oxidative stress [17], oral supplementation may not provide sufficient ongoing concentrations to achieve this. Uptake of oral vitamin C is highly regulated via intestinal vitamin C transporters (SVCTs), which limits the bioavailability of the vitamin at doses > 500 mg/day [31]. However, intravenous administration of the vitamin is able to bypass the regulated intestinal uptake, resulting in significantly higher plasma concentrations [23], with potentially improved efficacy. Although our oral vitamin C intervention was divided into two daily doses of 1 g/day, which improves bioavailability relative to a 2 g/day bolus dose, this may still not have been sufficiently bioavailable to counteract the enhanced utilisation of the vitamin due to the elevated inflammation and oxidative stress observed in these patients.

Furthermore, although we have previously shown that one week is sufficient time to raise plasma vitamin C concentrations to optimal in healthy individuals [32], a longer pre-supplementation period may be required for people with low vitamin C status to ensure adequate saturation of blood and tissues. A recent trial in 20 patients with myeloid cancer receiving 5-azacytidine who were supplemented with 500 mg/day vitamin C indicated that all patients, bar one, within the vitamin C arm reached ≥50 µmol/L plasma concentrations following one month of intervention [33]. However, another small study in six severely vitamin C depleted patients following allogeneic transplantation for leukaemia showed only 50% had reached ≥50 µmol/L plasma concentrations following one month of 2 g/day vitamin C supplementation orally [19]. Therefore, determinants of the optimal dosing regimen of vitamin C will include both the diagnosis and the chemotherapy regimen.

Oral mucositis is believed to be initiated by free radical damage which activates the inflammatory response [34]. Current treatment of oral mucositis is mainly supportive, e.g., ‘cryotherapy’ (ice chips in the mouth). Vitamin C could potentially attenuate the initial damage through detoxification of chemotherapy-induced oxidative stress and inflammation. In support of this premise, preliminary data suggests that patients with more severe mucositis tend to have lower vitamin C status [18], and supplementation with vitamin C has been shown to improve oral mucositis in a small group of patients suffering graft-versus-host disease following allogeneic transplantation for leukaemia [19]. Our cohort had a relatively low prevalence of patients with oral mucositis (40% at days 7 or 14; grade 1 or 2), and although those in the vitamin C group had no grade 2 oral mucositis, relative to two patients with grade 2 oral mucositis in the placebo group, the patient numbers were too low to determine if there was a significant difference between the groups. Nevertheless, further research on the effect of vitamin C intervention on oral mucositis appears warranted.

Our study was not intended to detect clinically meaningful differences in secondary clinical outcomes; however, we did observe significant inverse correlations between vitamin C status and various symptoms (nausea/vomiting, diarrhoea, fatigue, and pain) and positive associations with global health status and role and physical functioning. In addition to its antioxidant and anti-inflammatory functions, vitamin C has pleiotropic biosynthetic and gene regulatory enzyme cofactor functions which could contribute to its observed effects. For example, it plays an important role in the biosynthesis of amidated peptide hormones which have various regulatory functions in the gastrointestinal tract and other body systems [35,36]. The clinical data gathered as part of this study will provide reliable estimates of baseline values and patient variability in these outcomes that can be used for planning larger follow-up studies.

## 5. Conclusions

Overall, our study showed that whilst short-term pre-supplementation with oral vitamin C was able to restore adequate vitamin C status by day 0 in patients with myeloma and lymphoma, ongoing supplementation could not maintain adequate vitamin C concentrations following chemotherapy and transplantation. Thus, longer-term pre-supplementation may be required. However, due to issues with tolerance of, and subsequent compliance with, oral intervention in a number of the participants, intravenous vitamin C should be trialled as this bypasses the gastrointestinal system, negating intolerance issues and improving bioavailability of the vitamin.

## Figures and Tables

**Table 1:** Participant characteristics at baseline.

	Total Cohort (*n* = 20)	Placebo Group (*n* = 10)	Vitamin C Group (*n* = 10)
Age, years	55 (41, 64)	52 (41, 63)	59 (37, 68)
Gender, male	12 (60)	7 (70)	5 (50)
Weight, kg	79 (74, 84)	79 (75, 83)	77 (73, 93)
Smoker	5 (25)	3 (30)	2 (20)
Cancer type			
- myeloma - lymphoma	14 (70) 6 (30)	8 (80) 2 (20)	6 (60) 4 (40)
Myeloablative regimen			
- melphalan - BEAM ^1^ - carmustine + thiotepa	11 (55) 8 (40) 1 (5)	6 (60) 3 (30) 1 (10)	5 (50) 5 (50) 0 (0)

^1^ BEAM; carmustine + etoposide + cytarabine + melphalan. Data represent median (Q1, Q3) or *n* (%).

**Table 2:** Repeated measures correlations between vitamin C status and post-transplantation symptoms and functioning.

	RM Correlation (95% CI) *^a^*	*p* Value	≤23 µmol/L Vitamin C Subgroup	≥50 µmol/L Vitamin C Subgroup
**Infection parameter**				
C-reactive protein (mg/L)	−0.53 (−0.72, −0.26)	<0.001	47 (14, 123)	10 (4, 19)
**Gastrointestinal symptoms (%)**				
Nausea/vomiting	−0.52 (−0.71, −0.27)	<0.001	50 (17, 96)	17 (17, 33)
Diarrhoea	−0.53 (−0.72, −0.28)	<0.001	67 (33, 100)	33 (25, 42)
**Other symptoms (%)**				
Fatigue	−0.34 (−0.58, −0.05)	0.02	78 (61, 97)	56 (42, 92)
Pain	−0.40 (−0.63, −0.12)	0.006	33 (0, 46)	8 (0, 17)
**Functioning (%)**				
Global health status	0.43 (0.15, 0.65)	0.002	25 (17, 46)	50 (29, 67)
Role functioning	0.63 (0.40, 0.78)	<0.001	0 (0, 33)	42 (29, 67)
Social functioning	0.29 (−0.01, 0.54)	0.055	25 (0, 67)	50 (33, 67)
Physical functioning	0.42 (0.13, 0.64)	0.004	57 (27, 80)	67 (45, 77)

*^a^* Repeated measures (RM) correlations were carried out to take into account non-independence of observations. The total sample comprised 62 post-transplantation samples, the <23 µmol/L vitamin C subgroup comprised 24 samples, the ≥50 µmol/L vitamin C subgroup comprised 14 samples. The subgroup data are presented as median (Q1, Q3).

## Data Availability

Data is available upon reasonable request (e.g., for meta-analysis). The data are not publicly available due to ethical reasons.

## Supplementary Materials

The following are available online at https://www.mdpi.com/article/10.3390/antiox11101949/s1, Figure S1. Effect of oral supplementation on vitamin C status of participants. Placebo group, green symbols; vitamin C group, orange symbols. Dashed line indicates 50 μmol/L vitamin C. Data indicate mean and SEM. Mixed-effects analysis indicated a significant difference between the two groups; ** *p* = 0.002 by post-hoc analysis.
