# Supplementary material for: Supplementation with Oral Vitamin C Prior to and during Myeloablative Chemotherapy and Autologous Haematopoietic Stem Cell Transplantation: A Pilot Study
Source: Antioxidants (Basel). 2022 Sep 29;11(10):1949. doi: 10.3390/antiox11101949 (PMC9598083; doi:10.3390/antiox11101949)
Supplement: Supplementary file 1 [file antioxidants-11-01949-s001.zip › antioxidants-1875403-supplementary.pdf]

antioxidants

Supplemental material

Supplementation with Oral Vitamin C Prior to and During Myeloablative Chemotherapy and Autologous Haematopoietic Stem Cell Transplantation: a Pilot Study

Anitra C. Carr [1,*], Emma Vlasiuk [1], Masuma Zawari [1], Natalie Meijer [2], Carolyn Lauren [2], Sean MacPherson [2], Jonathan Williman [3] and Stephen Chambers [4]

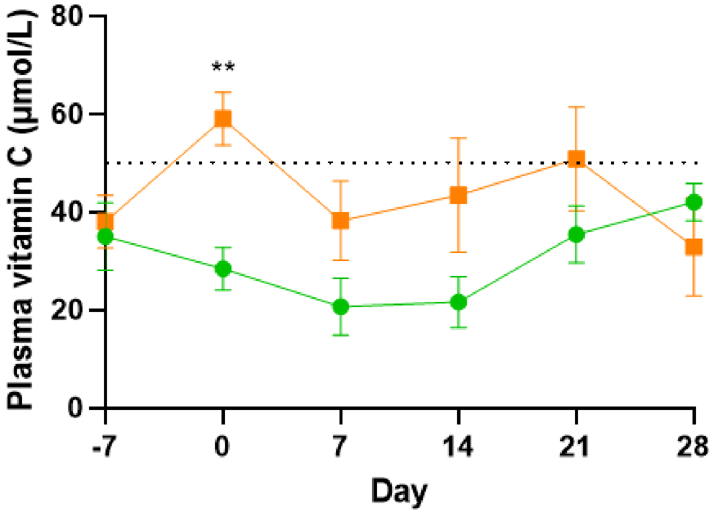

Figure S1. Effect of oral supplementation on vitamin C status of participants. Placebo group, green symbols; vitamin C group, orange symbols. Dashed line indicates 50 µmol/L vitamin C. Data indi-cate mean and SEM. Mixed-effects analysis indicated a significant difference between the two groups; ** p = 0.002 by post-hoc analysis.
